# Co-occurrence of non-alcoholic steatohepatitis exacerbates psoriasis associated with decreased adiponectin expression in a murine model

**DOI:** 10.3389/fimmu.2023.1214623

**Published:** 2023-08-14

**Authors:** Daiki Takezaki, Shin Morizane, Kenta Ikeda, Masanori Iseki, Yuma Sakamoto, Yoshio Kawakami, Taishi Hashiguchi, Yuka Shirakata, Sohji Nishina, Tomoyuki Mukai

**Affiliations:** ^1^ Department of Dermatology, Okayama University Graduate School of Medicine, Dentistry and Pharmaceutical Sciences, Okayama, Japan; ^2^ Department of Immunology and Molecular Genetics, Kawasaki Medical School, Okayama, Japan; ^3^ Department of Dermatology, National Hospital Organization Iwakuni Clinical Center, Yamaguchi, Japan; ^4^ SMC Laboratories, Inc, Tokyo, Japan; ^5^ Department of Gastroenterology and Hepatology, Kawasaki Medical School, Okayama, Japan

**Keywords:** psoriasis, non-alcoholic steatohepatitis, adiponectin, tumor necrosis factor-α, interleukin-17, epidermal keratinocyte

## Abstract

**Introduction:**

Clinical studies have suggested a bidirectional association between non-alcoholic steatohepatitis (NASH) and psoriasis, affecting each other’s development and severity. Here, we explored bidirectional causal linkages between NASH and psoriasis using a murine model.

**Methods:**

NASH was induced in mice by streptozotocin injection at 2 days of age and by high-fat diet feeding (STAM™ model). Psoriasis was induced by topical application of imiquimod (IMQ) on the ear. The severities of liver damage and psoriatic skin changes were determined using histological analysis. Gene expression in the skin tissues was evaluated using quantitative PCR analysis. Serum cytokine levels were determined using enzyme-linked immunosorbent assay. To examine the innate immune responses of normal human epidermal keratinocytes (NHEKs), the cells were treated with interleukin (IL)-17A, tumor necrosis factor (TNF)-α, and AdipoRon, an adiponectin receptor agonist.

**Results and Discussion:**

There were no differences in the degree of liver tissue damage (fat deposition, inflammation, and fibrosis) between NASH mice with and those without psoriasis. Conversely, the co-occurrence of NASH significantly augmented psoriatic skin changes, represented by epidermal hyperplasia, in psoriatic mice. Pro-inflammatory cytokines were expressed in the inflamed skin of psoriatic mice, and the expression of genes, especially *Il23a*, *Il1b*, *Il36g*, and *Mip2*, was significantly upregulated by the co-occurrence of NASH. The expression of keratinocyte activation marker genes *Defb4b* and *Krt16* was also upregulated by the co-occurrence of NASH. The serum TNF-α and IL-17 levels were increased by the co-occurrence of NASH and psoriasis. The serum adiponectin levels decreased in NASH mice compared with that in non-NASH mice. In NHEK culture, TNF-α and IL-17A synergistically upregulated *CXCL1*, *CXCL8*, and *IL1B* expression. The upregulated pro-inflammatory gene expression was suppressed by AdipoRon treatment, reflecting the anti-inflammatory capacity of adiponectin.

**Conclusion:**

The co-occurrence of NASH exacerbated psoriatic skin changes associated with increased serum inflammatory cytokine levels and decreased serum adiponectin levels. Combined with *in vitro* findings, increased inflammatory cytokine levels and decreased adiponectin levels likely promote innate immune responses in epidermal keratinocytes in psoriatic skin lesions. Overall, therapeutic intervention for co-occurring NASH is essential to achieve a favorable prognosis of psoriasis in clinical practice.

## 1 Introduction

Psoriasis is a chronic inflammatory skin disease characterized by thick, scaly, erythematous plaques; its global adult prevalence is approximately 2%–3% (1). Psoriasis is associated with obesity and metabolic diseases such as metabolic syndrome, dyslipidemia, and diabetes (2). Clinical studies have shown strong associations between obesity and psoriasis (3, 4). Both conditions increase the prevalence and severity of diseases bidirectionally (5). Metabolic disease-associated chronic inflammation is considered to activate a vicious cycle of inflammation. For instance, in our previous study, we found that obesity exacerbates psoriatic skin changes via increased leptin secretion and the subsequent innate immune responses of epidermal keratinocytes (6).

Non-alcoholic fatty liver disease (NAFLD) is a pathological liver condition affecting individuals who consume little to no alcohol. It ranges from intracellular fat deposition without inflammation (simple steatosis) to non-alcoholic steatohepatitis (NASH), which is accompanied by inflammation, cell damage, and fibrosis (7). The global prevalence of NAFLD and NASH is approximately 30% and 5%, respectively (8). NAFLD and NASH have become global health issues, as their prevalence has increased by 50% in the last 20 years (8).

NAFLD is a multisystem disease affecting various extrahepatic organs and increases the risk of cardiovascular disease, type 2 diabetes, and chronic kidney disease (9). Psoriasis is also recognized as an extrahepatic complication of NAFLD, with its prevalence increasing in patients with NAFLD (10, 11). Furthermore, the severity of psoriasis is higher in patients with NAFLD than in those without NAFLD (12). An inverse relationship has also been noted; patients with chronic psoriasis have nearly twice the odds of developing NAFLD compared to healthy controls (12). Bidirectional relationships between psoriasis and NAFLD have been suggested; however, the underlying causative relationships have not yet been clarified.

Adiponectin is an adipokine primarily secreted by adipose tissue (13). It plays a crucial role in regulating various physiological processes such as metabolism and inflammation (13). Adiponectin enhances the body’s sensitivity to insulin (13). Adiponectin also has anti-inflammatory properties (14, 15). It can suppress the production of pro-inflammatory cytokines and promote the secretion of anti-inflammatory cytokines (14, 15). Studies have shown that adiponectin levels are often decreased in patients with psoriasis (16, 17). This reduction in adiponectin level is suggested to contribute to persistent inflammation; however, the mechanisms are not fully elucidated. Therefore, in this study, we aimed to clarify the pathogenic links between NASH and psoriasis in a murine model focusing on adiponectin.

## 2 Materials and methods

### 2.1 Reagents

Imiquimod (IMQ) cream (Beselna Cream) was provided by Mochida Pharmaceutical (Tokyo, Japan). Streptozotocin (STZ) was purchased from Sigma-Aldrich Japan (Tokyo, Japan).

Recombinant human TNF-α, IL-17A, and AdipoRon were purchased from Chemicon (Temecula, CA, USA), R&D Systems (Minneapolis, MN, USA), and Focus Biomolecules (Plymouth Meeting, PA, USA), respectively.

### 2.2 Mice and diets

**Figure 1:**
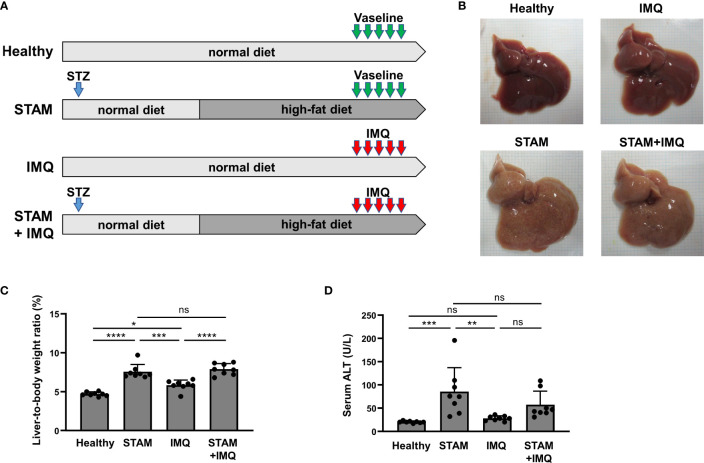
Generation of a murine model with both NASH and psoriasis. **(A)** Study protocol. Healthy mice were fed a normal diet (ND) for 11 weeks. Two-day-old male pups assigned to the STAM groups were subcutaneously injected with streptozotocin (STZ; 200 μg/mouse) on the back and fed the ND for 4 weeks until weaning, after which they were fed a high-fat diet (HFD) for 7 weeks. Imiquimod cream (IMQ; 25 mg/mouse) or Vaseline (as control) was topically applied to the ear of the mice at the age of 10 weeks for 5 consecutive days (*n* = 8 per group). Blood, liver, and skin samples were collected 1 day after the last treatment. **(B)** Photographs of the liver. **(C)** Liver-to-body weight ratio. **(D)** Serum alanine aminotransferase (ALT) level. Each dot denotes an individual mouse. Values are presented as mean with standard deviation. * *p* < 0.05, ** *p* < 0.01, *** *p* < 0.001, **** *p* < 0.0001 vs. indicated group. NASH, non-alcoholic steatohepatitis. ns, not significant.

Wild-type pregnant mice (C57BL/6J background) were purchased from Japan SLC, Inc. (Shizuoka, Japan) at 14 days of gestation. Neonatal male pups obtained after spontaneous parturition were used in this study. All mice were maintained at 23°C under a 12-h light/12-h dark cycle with free access to water and chow in SMC Laboratories, Inc. (Tokyo, Japan). All mice were fed a normal diet (ND; CE-2; CLEA Japan, Tokyo, Japan) for 4 weeks of weaning. After weaning, the healthy and IMQ group mice were fed ND, and the NASH (STAM) group mice were fed a high-fat diet (HFD; HFD32; CLEA Japan, Tokyo, Japan) (see **Figure 1A**). The mice were administered the diets until the end of the experiment.

### 2.3 NASH mouse model (STAM™ model)

To recapitulate the progression of NASH, we used a STAM™ murine model (18). Briefly, a single dose of STZ (200 µg/animal) was subcutaneously administered to the back of the mice at the age of 2 days to reduce insulin secretory capacity. The STAM mice were fed HFD from 4 weeks of age until the end of the experiments. The mice developed steatohepatitis at 8 weeks of age and progressive fibrosis at approximately 11 weeks of age, consistent with the development of NASH in mice (18, 19).

### 2.4 Imiquimod-induced psoriasis model

Psoriatic dermatitis was induced in mice by the topical application of IMQ, as described previously (6, 20). Mice were treated with 25 mg of 5% IMQ cream on the right ear for 5 consecutive days at the age of 10 weeks. Vaseline was used as a control. Ear thickness was measured daily using a digital thickness gauge. The mice were dissected 1 day after the last treatment. After euthanizing the mice, blood and tissue samples were collected.

STAM mice treated with IMQ were divided into four experimental groups, as shown in **Figure 1A**. For statistical power, we used eight mice per group based on our experience.

### 2.5 Histological analyses of the liver

After weighing the sampled liver, the left lobe was divided into three equal parts. The two pieces were fixed in Bouin’s fixative for 24 h, embedded in paraffin, and sectioned using a microtome. The sections were then subjected to hematoxylin–eosin (HE) and Sirius Red staining to evaluate intrahepatic fat deposition and fibrosis, respectively. The histological severity of steatohepatitis was evaluated using the NAFLD activity score, which was calculated according to Kleiner’s criteria based on images of HE-stained specimens (7). This score includes a numerical score for steatosis (0–3), hepatocyte ballooning (1–2), and lobular inflammation (0–3) (7).

Another piece of the liver was fixed in 10% formalin fixative for 24 h, immersed in sucrose solution, embedded in O.C.T. compound, and immediately frozen with liquid nitrogen. The frozen blocks were sectioned using a cryostat and subjected to Oil Red O staining. Five images per section were captured at a 200× field of view centered on the central vein. Oil Red O or Sirius Red staining-positive areas were measured using ImageJ (version 1.54d; NIH, Bethesda, MD, USA) (18).

### 2.6 Histological analyses of the ears

Histological analysis of the ear tissue was performed as described previously (21). The ear tissues were fixed in 10% formalin fixative for 24 h, embedded in paraffin, and sectioned using a microtome. The sections were stained with HE. Epidermal hyperplasia was quantified by measuring the thickness of the epidermis, excluding the stratum corneum, on the stained sections using ImageJ. Ten representative areas of the epidermis per mouse were measured, and the average values were calculated.

### 2.7 Real-time quantitative polymerase chain reaction analysis

Real-time quantitative polymerase chain reaction (qPCR) analysis was performed as described previously (22, 23). The total RNA was extracted from the right ear and liver using RNAiso Plus (Takara Bio, Shiga, Japan) and solubilized in ribonuclease-free water. Complementary DNA (cDNA) was synthesized using the PrimeScript RT Reagent Kit (Takara Bio). Real-time quantitative polymerase chain reaction was performed using the TB Green PCR Master Mix (Takara Bio) on the QuantStudio1 System (Thermo Fisher Scientific, Waltham, MA, USA). Gene expression levels were calculated using the ΔΔCt method and normalized to their levels in control samples indicated in each experiment. *36b4* was used as a housekeeping gene for murine samples and *GAPDH* was used for human epidermal keratinocyte samples. Real-time qPCR was performed using the primers listed in **Table S1**. All qPCRs yielded products with single-peak dissociation curves.

### 2.8 Measurement of biochemical parameters

Murine blood samples were centrifuged to separate the serum, and serum alanine aminotransferase (ALT) level was measured using a colorimetric method (GPT/ALT-PIII; FUJIFILM Co., Tokyo, Japan), following the manufacturer’s instructions.

### 2.9 Enzyme-linked immunosorbent assay

The levels of tumor necrosis factor (TNF)-α, interleukin (IL)-17, and adiponectin were measured in murine serum samples using the respective Quantikine enzyme-linked immunosorbent assay (ELISA) kits (R&D Systems) following the manufacturer’s protocol.

### 2.10 Cell culture and stimuli

Normal human epidermal keratinocytes (NHEKs) were obtained from Thermo Fisher Scientific (C-001-5C) and cultured in serum-free EpiLife Medium (Thermo Fisher Scientific) supplemented with 0.06 mM Ca^2+^ and EpiLife Defined Growth Supplement (Thermo Fisher Scientific) in 12-well flat-bottom plates at 37°C with 5% CO_2_. The cells were maintained for a maximum of eight passages in this medium supplemented with penicillin (100 IU/mL), streptomycin (100 µg/mL), and amphotericin B (0.25 µg/mL). When the cells reached 70% confluence, they were stimulated with IL-17A (30 ng/mL) and TNF-α (30 ng/mL) for 24 h. Thereafter, AdipoRon (10, 30, 100, and 300 μM) was added 30 min before or 60 min after IL-17A and/or TNF-α treatment. RNA samples were then collected using RNAiso Plus and used for qPCR analysis.

### 2.11 Ethical approval

All animal experiments were approved by the Institutional Animal Care and Use Committee of SMC Laboratories, Inc. (SLMN#043-2209-4). All experimental procedures were conducted in accordance with the institutional and NIH guidelines for the humane use of animals.

### 2.12 Statistical analysis

All values are presented as mean with standard deviation, except the values of the time course of the ear thickness, which are presented as mean ± standard error of the mean. A one-way analysis of variance, followed by Tukey’s *post-hoc* test, was used to compare three or more groups. All statistical analyses were performed using GraphPad Prism 9 (GraphPad Software, San Diego, CA, USA). Statistical significance was set at *p* < 0.05.

## 3 Results

### 3.1 Generation of the murine model with both NASH and psoriasis

To investigate the bidirectional pathogenic links between NASH and psoriasis, we generated a murine model with both NASH and psoriasis. NASH was induced by STZ injection and HFD feeding (STAM model), and psoriasis was induced by the topical application of IMQ to the ear. The mice were divided into four groups, as shown in **Figure 1A**.

The livers isolated from STAM group mice were yellow (**Figure 1B**). The liver-to-body weight ratio of STAM group mice was higher than that of healthy and IMQ mice (**Figure 1C**). The serum ALT level of STAM group mice was higher than that of healthy and IMQ mice (**Figure 1D**).

### 3.2 Effects of psoriasis on the severity of NASH

**Figure 2:**
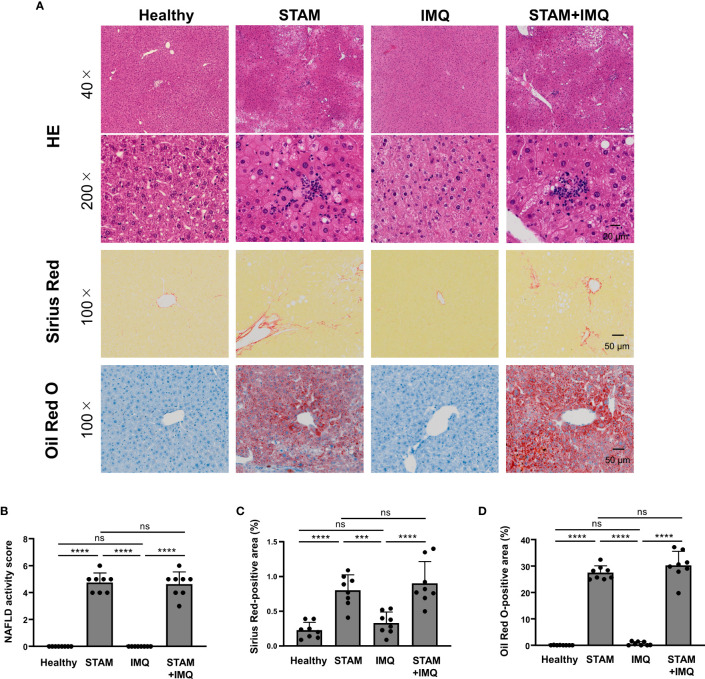
Histological analyses of the liver. Non-alcoholic steatohepatitis (NASH) and psoriatic skin changes were induced as described in Methods and **Figure 1**. Liver tissues were collected 1 day after the last treatment. **(A)** Representative images of the liver stained by hematoxylin–eosin, Sirius Red, and Oil Red O. **(B)** Non-alcoholic fatty liver disease (NAFLD) activity score according to Kleiner’s criteria. **(C, D)** Percentage of Sirius Red- and Oil Red O-stained areas. Each dot denotes an individual mouse. Values are presented as mean with standard deviation. *** *p* < 0.001, **** *p* < 0.0001 vs. indicated group. ns, not significant.

To examine whether psoriasis affects the severity of NASH, we performed liver histological analyses. The liver specimens of STAM mice showed balloon-like degeneration of hepatocytes, deposition of large and small fat droplets, and infiltration of inflammatory cells, including lymphocytes and neutrophils (**Figure 2A**), which were comparable to the pathology of human NASH. IMQ-induced psoriasis did not augment the severity of NASH, as indicated by the NAFLD activity scores (**Figure 2B**). Moreover, psoriasis did not enhance the severity of liver fibrosis and fat deposition (**Figures 2C, D**).

### 3.3 Effects of NASH on the severity of psoriasis

**Figure 3:**
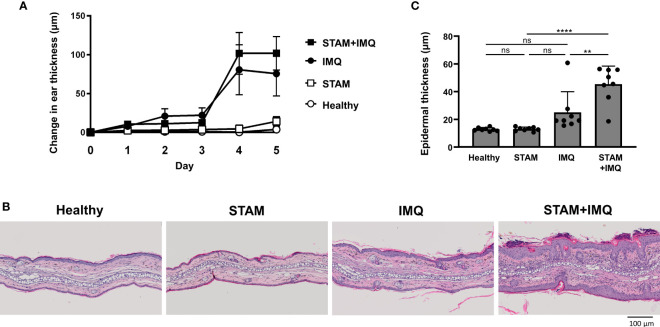
Co-occurrence of NASH exacerbated psoriatic skin changes. **(A)** Changes in ear thickness. Mice were topically treated with imiquimod (IMQ) cream (25 mg/mouse) or Vaseline (control; *n* = 8 per group). The ear thickness of each mouse was measured using a digital thickness gauge daily. Values are presented as mean ± standard error of the mean. **(B)** Representative images of hematoxylin–eosin-stained ears of indicated mice. Bar = 100 μm. **(C)** Histological analysis of epidermal thickness. Ten representative areas of the epidermis were measured for individual mice, and their average value was calculated. Each dot denotes an individual mouse. Values are presented as mean with standard deviation. ** *p* < 0.01, **** *p* < 0.0001 vs. indicated group. NASH, non-alcoholic steatohepatitis. ns, not significant.

Next, we investigated whether the presence of NASH affects the severity of psoriasis; for this purpose, the development of psoriatic lesions was examined after topical IMQ treatment. The IMQ treatment aggravated skin thickening over time, and the co-occurrence of NASH tended to worsen skin thickening (**Figure 3A**). In the Vaseline-treated group, the co-occurrence of NASH did not significantly affect ear thickness (**Figure 3A**). In the histological analysis, we found epidermal thickening and inflammatory cell infiltrates in the skin of IMQ-treated mice (**Figure 3B**). Additionally, the co-occurrence of NASH significantly enhanced epidermal thickness, parakeratosis, and inflammatory cell infiltrates in psoriatic mice (**Figure 3B**). The exacerbated epidermal thickness in IMQ-treated STAM mice was confirmed by the quantitative analysis of the skin (**Figure 3C**).

### 3.4 Enhanced pro-inflammatory cytokine expression in the inflamed skin by NASH

**Figure 4:**
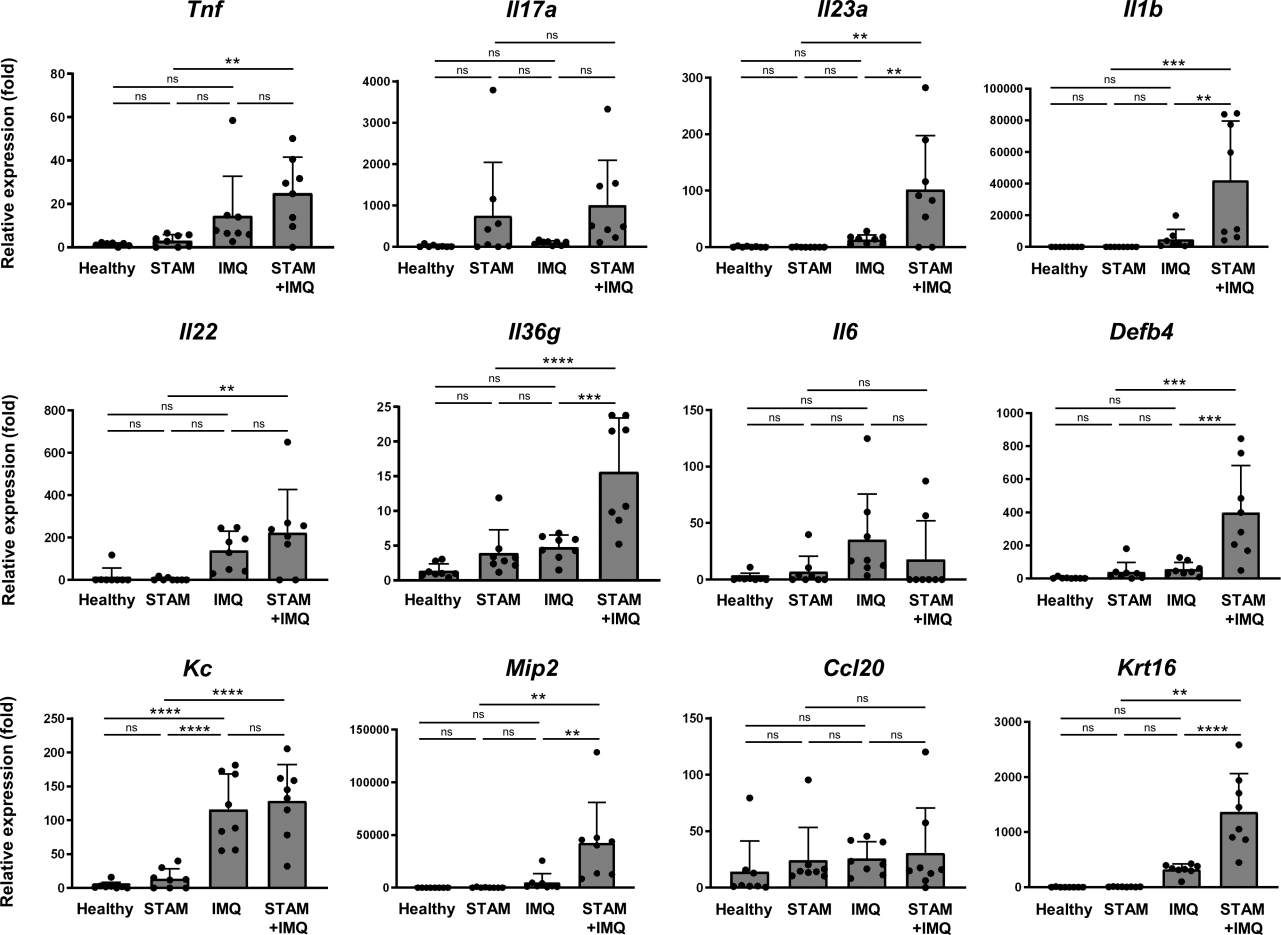
Quantitative polymerase chain reaction analysis of the ear. Alterations in the mRNA expression of pro-inflammatory cytokines associated with psoriasis. RNA samples were extracted from the ear tissues of the indicated mice. mRNA expression levels were determined using quantitative PCR analysis. The levels were calculated relative to the level of *36b4* and normalized to the expression level in the healthy group. Each dot denotes an individual mouse. Values are presented as mean with standard deviation. ** *p* < 0.01, *** *p* < 0.001, **** *p* < 0.0001 vs. indicated group. IMQ, imiquimod. ns, not significant.

To elucidate the mechanisms by which accompanying NASH exacerbates psoriatic skin phenotypes, we assessed the expression of pro-inflammatory cytokine genes in inflamed skin lesions. The expression of pro-inflammatory cytokine was moderately upregulated in IMQ-treated mice compared with that in healthy mice (**Figure 4**). Interestingly, the co-occurrence of NASH significantly upregulated the gene expression of *Il23a*, *Il1b*, *Il36g*, and *Mip2*, compared with that in IMQ-treated mice without NASH (**Figure 4**). In addition, the expression of the antimicrobial peptide gene *Defb4* and the keratinocyte proliferation marker *Krt16* was upregulated by the co-occurrence of NASH (**Figure 4**).

Notably, in the groups not treated with IMQ, the co-occurrence of NASH moderately increased the expression of some genes, such as *Il17a* and *Il36g* (**Figure 4**), although the changes were not significant owing to the large variation in the data.

### 3.5 Altered serum cytokine levels by NASH

**Figure 5:**
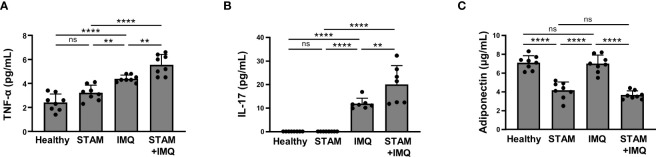
Serum cytokine levels. Blood samples were collected from the indicated mice treated with imiquimod (IMQ) cream (25 mg/mouse) or Vaseline (control; *n* = 8 per group). **(A–C)** Serum levels of tumor necrosis factor (TNF)-α, interleukin (IL)-17, and adiponectin were measured using enzyme-linked immunosorbent assay (ELISA). Each dot denotes an individual mouse. Values are presented as mean with standard deviation. *** *p* < 0.001, **** *p* < 0.0001 vs. indicated group. ns, not significant.

Psoriasis and NAFLD have common feature of chronic inflammation, and both exert systemic effects. We next evaluated the systemic inflammatory factors that account for the exacerbation of psoriasis in NASH mice. The serum levels of TNF-α and IL-17 were synergistically elevated by the co-occurrence of NASH and psoriasis (**Figures 5A, B**).

Notably, the serum adiponectin level significantly decreased in NASH mice compared with that in non-NASH mice (**Figure 5C**).

### 3.6 Suppressive effect of adiponectin on the pro-inflammatory response in epidermal keratinocytes

The reduced serum adiponectin level in NASH mice led us to speculate about the involvement of adiponectin in the pathogenesis of exacerbated skin inflammation in IMQ-treated STAM mice. To investigate the potential involvement of adiponectin, we evaluated the effect of adiponectin on epidermal keratinocytes. Epidermal keratinocytes act as initiator and amplifier cells in the pathogenesis of psoriasis through the production of chemokines and inflammatory cytokines (24). In terms of the importance of epidermal keratinocytes in the pathology of psoriasis, we have previously clarified the essential roles of epidermal keratinocytes in psoriasis (6, 25–27).

**Figure 6:**
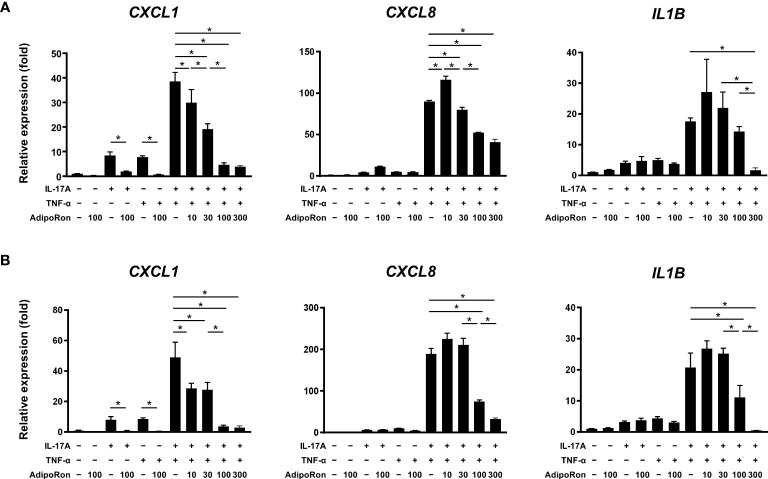
AdipoRon suppressed innate immune responses of epidermal keratinocytes. Normal human epidermal keratinocytes were stimulated with interleukin (IL)-17A (30 ng/mL) and tumor necrosis factor (TNF)-α (30 ng/mL) for 24 h. AdipoRon (10, 30, 100, and 300 μM) was added 30-min before **(A)** or 60-min after **(B)** the IL-17A and/or TNF-α treatment. Gene expression levels relative to *GAPDH* level were calculated and normalized to the levels in the non-stimulated control. Values are presented as mean with standard deviation. * *p* < 0.05 vs. indicated group.

Cultured NHEKs were treated with AdipoRon, an adiponectin receptor agonist, in combination with IL-17A and/or TNF-α. We found that treatment with IL-17A, TNF-α, and their combination significantly upregulated the expression of *CXCL1*, *CXCL8*, and *IL1B* (**Figure 6A**). AdipoRon pretreatment significantly suppressed the pro-inflammatory gene expression (**Figure 6A**). In addition, 60 min after treatment of AdipoRon significantly suppressed the gene expression to levels similar to those of the pretreatment experiment (**Figure 6B**). These findings reflect the anti-inflammatory nature of adiponectin (13).

## 4 Discussion

In this study, the co-occurrence of NASH significantly augmented epidermal hyperplasia in psoriatic mice, which was associated with decreased serum adiponectin levels. Adiponectin is an adipokine produced by adipose tissue, and its secretion is reduced by obesity (13). It serves as an anti-inflammatory cytokine (13). In the present study, the serum adiponectin level significantly decreased in NASH mice. In *in vitro* experiments using NHEKs, stimulation of adiponectin receptors significantly suppressed TNF-α- and IL-17A-induced pro-inflammatory cytokine gene expression. These findings suggest that the presence of NASH reduces adiponectin production, which in turn decreases the anti-inflammatory effect of adiponectin, resulting in exacerbated psoriatic skin changes, at least partially, by directly acting on epidermal keratinocytes. Supporting this notion, a previous clinical study reported associations between psoriasis and decreased serum adiponectin levels (28–30). Additionally, an earlier study in a murine model has shown the involvement of adiponectin in the pathogenesis of psoriasis (31). In a murine model, adiponectin deficiency exacerbated psoriasiform dermatitis by promoting the infiltration of IL-17-producing dermal γδT cells (31). Therefore, adiponectin is suggested to contribute to the exacerbation of dermatitis in patients with psoriasis as a NASH-associated systemic factor.

In addition to adiponectin, pro-inflammatory cytokines are likely to contribute to the worsening of psoriatic skin changes as NASH-associated systemic factors. NASH is known to exert systemic effects, leading to the development or exacerbation of cardiovascular diseases, type 2 diabetes, chronic kidney disease, and hypothyroidism (32). In terms of mechanism, the involvement of intrahepatic Th17 cells and serum IL-6, IL-17, and IL-23 has been demonstrated in a murine NASH model and human patients with NAFLD (33, 34). The serum levels of TNF-α and IL-6 are elevated in patients with NASH and are associated with disease severity (35, 36). As inflammatory cytokines are also critically involved in the pathogenesis of psoriasis, NASH-associated inflammatory cytokines are likely to be exacerbating factors of psoriatic skin changes. This notion is supported by our *in vitro* findings, showing that IL-17A and TNF-α augment innate immune responses of epidermal keratinocytes, represented by increased chemokine and inflammatory cytokine production.

The effect of psoriasis on the development or severity of NAFLD remains controversial in clinical practice. Some studies have reported a higher prevalence and more severe phenotypes of NAFLD in patients with psoriasis (37, 38), while others have found no significant association between psoriasis and liver fibrosis (39, 40). For instance, the risk of patients with new-onset psoriasis developing NAFLD in the future is 1.28-fold higher than that of patients without psoriasis (11). Besides, a meta-analysis showed that 9.66% of patients with psoriasis were at an increased risk of developing advanced liver fibrosis (39). Although the co-occurrence of psoriasis did not significantly augment the severity of liver damage in our experimental model, the findings need to be re-evaluated by using other murine models with prolonged psoriasis. As the psoriatic skin phenotypes are induced in a short period (5 days) in the IMQ-induced psoriatic model, the disease duration may have been too short to worsen NAFLD pathology. Therefore, other murine models that present prolonged psoriasis, such as genetically modified psoriasis models (41, 42), would be required to evaluate the effects of chronic psoriasis on the development or progression of NAFLD.

Interestingly, we found that the co-occurrence of NASH moderately increased *Il17a* and *Il36g* expression in the skin tissues without IMQ treatment, although the changes did not reach significance. This observation suggests that NASH can induce subclinical skin inflammation without direct skin stimulation. In our previous study, we found that aberrant metabolic conditions, obesity and dyslipidemia, induce characteristic expression patterns of pro-inflammatory cytokines in the skin (6). HFD feeding tended to elevate *Ccl20* expression in the skin, whereas *Apoe* deficiency-mediated dyslipidemia upregulated *Il19* expression in the skin (6). Another group reported similar findings, indicating that short-term exposure to a Western diet composed of high fat content and simple sugars induced psoriasiform dermatitis in mice (43). Thus, our current and previous study findings suggest that aberrant metabolic conditions predispose the skin to psoriasis, a so-called pre-psoriatic state.

In addition to adiponectin, other factors have been suggested to be involved in the liver–skin axis. Leucine-rich α-2 glycoprotein (LRG) modulates the liver–skin axis and is involved in the pathogenesis of psoriasiform inflammation (44). LRG deficiency resulted in mild psoriatic skin changes associated with decreased inflammatory cytokine expression in a murine model of psoriasis. Thus, clarifying the detailed mechanisms of the liver–skin axis in psoriasis is necessary to develop novel treatments.

There may be some possible limitations to this study. A more comprehensive analysis would be required to comprehensively clarify the pathological link between psoriasis and NASH. Although we have shown the possible involvement of adiponectin, the effects of factors related to metabolic abnormalities on skin inflammation are diverse and complex. Various metabolic disease-associated factors other than adiponectin have been reported to affect psoriatic skin phenotypes, such as leptin (6, 45), chemerin (46), resistin (16), and free fatty acids (6, 47). Additionally, metabolic disease-associated factors could directly act on immune cells, including macrophages (48), lymphocytes (15, 49), and innate lymphoid cells (50, 51), not just on epidermal keratinocytes.

Another concern is that STZ-induced hyperglycemia and HFD may have acted as confounding factors to the exacerbation of psoriasis. Two groups have reported that STZ and HFD-induced diabetic mice exhibit more severe psoriatic skin changes after IMQ treatment than non-diabetic control mice (52, 53). Although the timing of STZ injection and duration of HFD are different between their study and our study, their findings suggest that STZ-induced hyperglycemia and/or HFD could affect the severity of psoriatic skin changes.

In conclusion, our findings suggest that the co-occurrence of NASH exacerbates psoriasis associated with decreased serum adiponectin level. Further research is required to clarify possible causal relationships between decreased adiponectin level and psoriasis exacerbation. Such studies could help develop effective treatment strategies for psoriasis accompanying NAFLD.

## Data availability statement

The raw data supporting the conclusions of this article will be made available by the authors, without undue reservation.

## Ethics statement

Ethical approval was not required for the studies on humans in accordance with the local legislation and institutional requirements because only commercially available established cell lines were used. The animal study was approved by the Institutional Animal Care and Use Committee of SMC Laboratories, Inc. The study was conducted in accordance with the local legislation and institutional requirements.

## Author contributions

DT, KI, TH, and YSh performed the experiments and analyzed the data. SM, MI, YSa, YK, SN, and TM helped in the interpretation of the data. DT and TM wrote the manuscript. All authors read and approved the manuscript.
