# Supplementary material for: Co-occurrence of non-alcoholic steatohepatitis exacerbates psoriasis associated with decreased adiponectin expression in a murine model
Source: Front Immunol. 2023 Aug 14;14:1214623. doi: 10.3389/fimmu.2023.1214623 (PMC10461570; doi:10.3389/fimmu.2023.1214623)
Supplement: Supplementary file 1 [file Table_1.docx]

**Supplemental Table 1 qPCR primers used in this study**

| Gene | Gene accession number | Sequence | | Amplicon  size (bp) | Annealing temperature (°C) |
| --- | --- | --- | --- | --- | --- |
| *Tnf* | NM_013693.2 | Forward:  Reverse: | catcttctcaaaattcgagtgaca  tgggagtagacaaggtacaaccc | 175 | 60 |
| *Il17a* | NM_010552.3 | Forward:  Reverse: | tgtgaaggtcaacctcaaagtc  gagggatatctatcagggtcttca | 132 | 60 |
| *Il23a* | NM_031252.2 | Forward:  Reverse: | caccagcgggacatatgaa  ccttgtgggtcacaaccat | 100 | 60 |
| *Il1b* | NM_008361.3 | Forward:  Reverse: | agttgacggaccccaaaag  agctggatgctctcatcagg | 75 | 60 |
| *Il22* | NM_016971.2 | Forward:  Reverse: | tgacgaccagaacatccaga  aatcgccttgatctctccac | 85 | 60 |
| *Il36g* | NM_153511.3 | Forward:  Reverse: | ggacaccctactttgctgcta  aacaggaatggcttcattgg | 80 | 60 |
| *Il6* | NM_031168.1 | Forward:  Reverse: | aacgatgatgcacttgcaga  ccagaggaaattttcaataggc | 113 | 60 |
| *Defb4* | NM_019728.4 | Forward:  Reverse: | cagtcatgaggatccattacctt  aatttgggtaaaggctgcaa | 77 | 60 |
| *Kc* | NM_008176.3 | Forward:  Reverse: | cttgaccctgaagctccctt  gttgtcagaagccagcgttc | 127 | 60 |
| *Mip2* | NM_009140.2 | Forward:  Reverse: | aaaatcatccaaaagatactgaacaa  ctttggttcttccgttgagg | 91 | 60 |
| *Ccl20* | NM_016960.2 | Forward:  Reverse: | aactgggtgaaaagggctgt  gtccaattccatcccaaaaa | 86 | 60 |
| *Krt16* | NM_001313958.1 | Forward:  Reverse: | atgagctgaccctgtccaga  ctcaaggcaagcatctcctc | 106 | 60 |
| *36b4* | NM_007475.5 | Forward:  Reverse: | tcctcgttggagtgacatcg  tagttggacttccaggtcgc | 98 | 60 |
| *CXCL1* | NM_001511.3 | Forward:  Reverse: | catcgaaaagatgctgaacagt  ataagggcagggcctcct | 94 | 60 |
| *CXCL8* | NM_000584.3 | Forward:  Reverse: | cagagacagcagagcacaca  gcactccttggcaaaactgc | 167 | 60 |
| *IL1B* | NM_000576.2 | Forward:  Reverse: | aaagcttggtgatgtctggtc  ggacatggagaacaccacttg | 89 | 60 |
| *GAPDH* | NM_002046.5 | Forward:  Reverse: | agccacatcgctcagacac  aatacgaccaaatccgttgact | 62 | 60 |
